# Long-Term Outcomes following Cytoreductive Surgery and Hyperthermic Intraperitoneal Chemotherapy for Peritoneal Carcinomatosis of Colorectal Origin

**DOI:** 10.3390/curroncol31070269

**Published:** 2024-06-25

**Authors:** Kadhim Taqi, Jay Lee, Scott Hurton, Cecily Stockley, Lloyd Mack, Justin Rivard, Walley Temple, Antoine Bouchard-Fortier

**Affiliations:** 1 Division of Surgical Oncology, Department of Oncology, Cumming School of Medicine, University of Calgary, Calgary, AB T2N 1N4, Canada; 2 Department of Surgery, Cumming School of Medicine, University of Calgary, Calgary, AB T2N 1N4, Canada; 3 Division of Surgery, Max Rady College of Medicine, University of Manitoba, Winnipeg, MB R3E 0W2, Canada

**Keywords:** colorectal cancer, cytoreductive surgery (CRS), HIPEC, colorectal metastasis, peritoneal carcinomatosis, long-term survival

## Abstract

Background: Cytoreductive surgery (CRS) combined with hyperthermic intraperitoneal chemotherapy (HIPEC) is a major treatment of colorectal peritoneal carcinomatosis (CPC). The aim was to determine the disease-free survival (DFS) and overall survival (OS) of patients undergoing CRS–HIPEC for CPC and factors associated with long-term survival (LTS). Methods: consecutive CPC patients who underwent CRS–HIPEC at a HIPEC center between 2007 and 2021 were included. Actual survival was calculated, and Cox proportional hazards models were used to identify factors associated with OS, DFS and LTS. Results: there were 125 patients with CPC who underwent primary CRS–HIPEC, with mean age of 54.5 years. Median follow-up was 31 months. Average intraoperative PCI was 11, and complete cytoreduction (CC-0) was achieved in 96.8%. Median OS was 41.6 months (6–196). The 2-year and 5-year OS were 68% and 24.8%, respectively, and the 2-year DFS was 28.8%. Factors associated with worse OS included pre-HIPEC systemic therapy, synchronous extraperitoneal metastasis, and PCI ≥ 20 (*p* < 0.05). Progression prior to CRS–HIPEC was associated with worse DFS (*p* < 0.05). Lower PCI, fewer complications, lower recurrence and longer DFS were associated with LTS (*p* < 0.05). Conclusion: CRS and HIPEC improve OS in CPC patients but they have high disease recurrence. Outcomes depend on preoperative therapy response, extraperitoneal metastasis, and peritoneal disease burden.

## 1. Introduction

Peritoneal carcinomatosis occurs in approximately 5–10% of patients with colorectal adenocarcinoma, and often carries poor prognosis [1,2]. Various studies have demonstrated improved overall survival (OS) with cytoreductive surgery and hyperthermic intraperitoneal chemotherapy (CRS–HIPEC) over systemic therapy alone [1,2,3,4]. Long-term survival (LTS) after CRC–HIPEC for colorectal peritoneal carcinomatosis (CPC) have an estimated 5-year OS between 19–35%, which is in stark contrast to systemic treatment alone [2,3,4]. The median OS ranges between 22 and 60 months while the median disease-free survival (DFS) is lower (9–16 months), reflecting the high rates of disease recurrence [1,2,3,5,6,7]. The difference observed in the range of OS and DFS is attributed to various factors including peritoneal carcinomatosis index (PCI), completeness of cytoreduction (CC), lymph node positivity, and postoperative surgical complications [6]. CRS–HIPEC can be associated with significant morbidity and therefore requires astute patient selection to derive the greatest benefit [7].

Most retrospective studies provide estimated LTS in patients who undergo CRS–HIPEC for CPC, but few have reported on actual LTS or those who may be potentially cured of their disease. Currently, the proportion of patients who survive long term after CRS–HIPEC for CPC is unknown in Alberta, Canada. The primary objective of this study was to determine the OS and DFS for patients treated with CRS–HIPEC for CPC, and factors associated with outcome and LTS.

## 2. Materials and Methods

This is a retrospective study of all consecutive patients who underwent CRS–HIPEC for CPC from a single HIPEC center. Patients were eligible for analysis if they had confirmed colorectal adenocarcinoma pathology and underwent primary CRS–HIPEC. All data were collected as part of a database in the Division of Surgical Oncology at the Tom Baker Cancer Centre between January 2007 and December 2021. This database is approved through the University of Calgary Ethics Board and includes all patients with peritoneal carcinomatosis.

During this time, CRS–HIPEC was performed by 3 surgeons in our center. All procedures were performed open. PCI was calculated intraoperatively. Patients were excluded if they did not undergo CRS–HIPEC. Reasons for aborted CRS–HIPEC included unresectable portal triad involvement, significant small bowel involvement (<100 cm small bowel remaining), unexpected/unresectable liver or retroperitoneal involvement, or if CC was not possible due to unresectable structures. From January 2007 to January 2008, patients with a CC-0 or CC-1 score were treated with a standardized protocol consisting of HIPEC with mitomycin C (MMC) (12–15 mg in 3 L of dianeal, circulated for 60 min at 40–42 °C), followed by early postoperative intraperitoneal chemotherapy (EPIC) with Fluorouracil (5-FU) (1000 mg daily for 24 h on postoperative day 1 through 5). Beginning in February 2008, the protocol was changed to HIPEC with oxaliplatin (400 mg in 3 L of 5% glucose, circulated for 60 min at 40–42 °C) with a simultaneous dose of intravenous 5-FU (800 mg). This was done as EPIC was dropped due to increased morbidity and Oxaliplatin became more available in the province. Oxaliplatin was switched back to MMC 40 mg in 3 L dianeal again after the publication of PRODIGE-7 [7]. Patients were most often treated with 6–12 cycles of perioperative systemic chemotherapy (FOLFOX or FOLFIRI with or without Bevacizumab) with assessment of response on subsequent imaging.

All data were extracted from the Alberta Cancer Registry and the Cancer Surgery Alberta synoptic reporting system (Synoptec^®^, Irvine, CA, USA). Demographics, index cancer, surgical and postoperative data were collected. Race and ethnicity details were not recorded as part of the database. Follow-up data were obtained through consultation notes or follow-up imaging. During this period, the University of Calgary was a major referral center for all provinces between and including Ontario and British Columbia. Patients received CRS–HIPEC in Calgary but had subsequent follow-up and treatment in their referring provinces.

Descriptive statistics were calculated, and continuous variables were compared using the Mann–Whitney U test, while categorical variables were compared using Pearson chi-square or Fisher’s exact test. Kaplan–Meier curves were produced, using the log-rank test to determine differences between groups. OS was calculated from the date of carcinomatosis diagnosis to the date of death, and DFS from the date of CRS–HIPEC to the date of the first evidence of local or distant recurrence, whether clinically or on imaging. Univariate and multivariate analyses were performed using Cox regression modelling. Variables were selected for analysis based on previous literature and clinically relevant prognostic factors. Patients were categorized into the LTS group if they were alive ≥5 years from the time of CRS–HIPEC, and in the non-LTS group if their survival was <5 years. All analyses were performed using the Statistical Package for the Social Sciences ^®^ (SPSS, version 23.0, Chicago, IL, USA).

## 3. Results

A total of 125 patients underwent primary CRS–HIPEC for CPC from January 2007 to December 2021, with a median follow-up of 31 months (5–198). The mean age was 54.5 years (23.5–76.1), with 50.4% (n = 63/125) male patients. Most patients (90.4%, n = 113/125) had prior surgery for the index colorectal cancer, which was mostly located in the sigmoid colon (33.6%, n = 42/125) and the ascending colon (26.4%, n = 33/125). The majority of patients (65.6%, n = 82/125) had a node-positive index colorectal cancer, while the pathology showed mucinous type adenocarcinoma in 28% (n = 35/125), and 8% (n = 10/125) were BRAF mutated. Table 1 summarizes the demographic and index cancer characteristics.

Synchronous peritoneal metastasis was the initial presentation in 65 patients (52%), while 17.6% (n = 22/125) had further liver or ovarian metastasis as well (intra-parenchymal extraperitoneal metastasis). The majority of patients (80.8%, n = 101/125) received preoperative systemic therapy between 6 and 12 cycles of FOLFOX or FOLFIRI with or without bevacizumab as per the center’s protocol. A total of 24 patients underwent upfront surgery for low PCI (median 8), symptomatology (n = 6) or toxicity related to chemotherapy (n = 3). Response to systemic therapy was assessed using subsequent computed tomography (CT) scan or positron emission tomography (PET) scan. Radiological response (complete or partial) was seen in 46.5% (n = 47/125), while 40 patients had stable disease (39.6%) and 14 patients (13.9%) had progression of disease. CC-0 was achieved in 121 patients (96.8%), and the median PCI was 11 (0–39). EPIC was used in 8 patients, and 20 patients received MMC, while intraperitoneal oxaliplatin with intravenous 5-FU was used in 105 patients. The average length of hospital stay was 20.9 days (7–84) and 21 patients (16.8%) required ICU admission postoperatively. Seventy-five patients (60%) had postoperative complications with Clavien–Dindo Grade III and IV complications seen in 19 (25.3%) and 3 (4%) patients, respectively. The most common complications were postoperative ileus (n = 24), surgical site infection (n = 15), intra-abdominal collection (n = 11) and deep venous thrombosis (n = 6). Anastomosis leak (n = 5) and wound dehiscence (n = 2) were the most common reasons for reoperation. There were no perioperative mortalities in the cohort. Table 1 demonstrates the perioperative characteristics.

**Figure 1:**
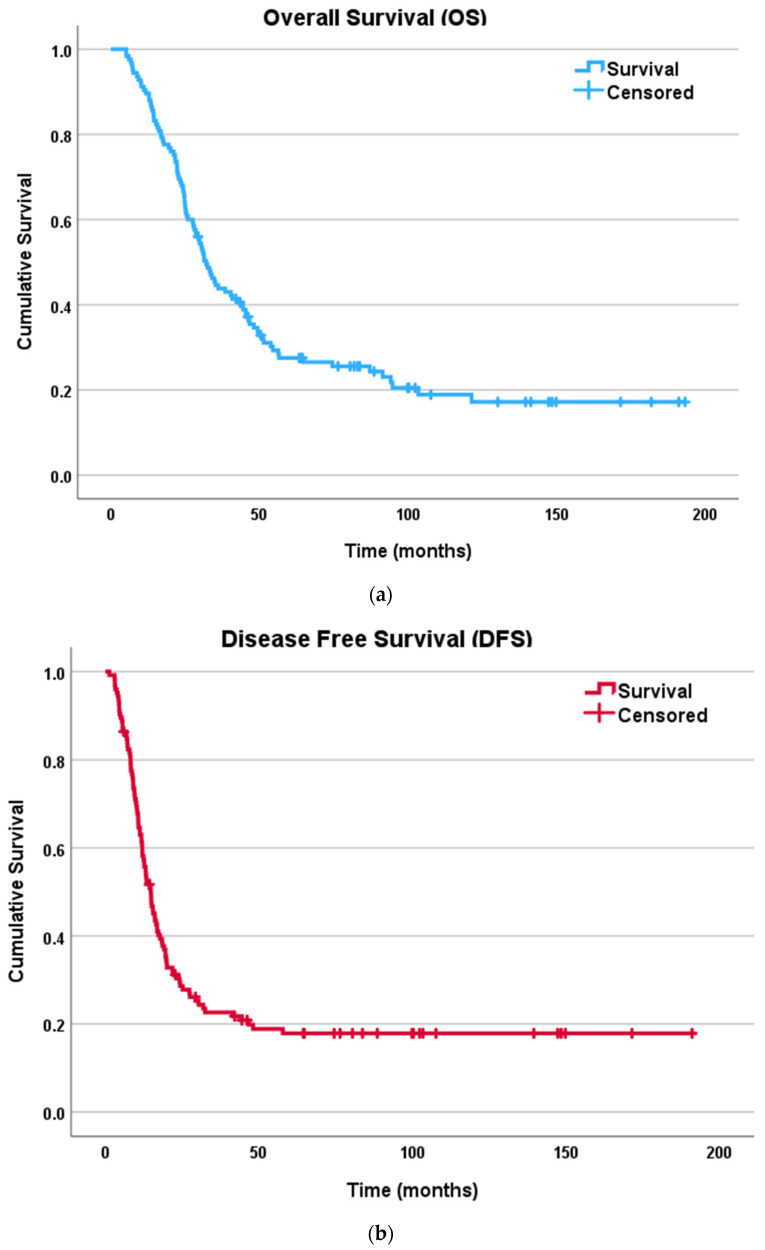
Kaplan–Meier survival curves for patients undergoing CRS–HIPEC for colorectal peritoneal carcinomatosis: (**a**) overall survival (OS); (**b**) disease free survival (DFS).

The 2-year OS for the entire population was 68% while the 5-year OS was 24.8%. The median OS was 41.6 months (6–196) from the time of carcinomatosis diagnosis. A total of 18 patients (14.4%) were completely disease-free at the study end date. The recurrence rate was 80% with average disease-free interval of 29.3 months and median time to recurrence of 14.5 months (1–191). The 2-year DFS was 28.8% while the 5-year DFS was 14.4%. Most of the recurrences occurred in the peritoneum (42%, n = 42/100) with various disease severity and retroperitoneal involvement, followed by peritoneum and liver (12%, n = 12/100) and peritoneum and lung (11%, n = 11/100). Figure 1 demonstrates the Kaplan–-Meier curves with OS and DFS of the entire population.

On univariate analysis, significant associations were found between OS and: high PCI (≥20) (HR = 1.85, 95% CI: 1.12–3.06, *p* = 0.017), synchronous extraperitoneal metastasis (HR = 1.13, 95% CI 1.08–1.90, *p* = 0.039), and pre-HIPEC systemic therapy (HR 1.49, 95% CI 1.19–2.49, *p* = 0.032). Progression on pre-HIPEC systemic therapy was significantly associated with DFS (HR 1.57, 95% CI 1.38–2.81, *p* = 0.043). On multivariate analysis, OS continued to be associated with PCI ≥ 20 (HR = 1.92, 95% CI: 1.65–5.66, *p* = 0.01). Age, gender, index cancer origin and nodal status, preoperative systemic therapy, response to systemic therapy, extraperitoneal metastasis, synchronous peritoneal presentation, and post-HIPEC systemic therapy did not demonstrate significant association with OS and DFS on multivariate analysis. CC score was not included in the analysis as the numbers were small for CC-1 and CC-2. Given the long duration of the study and the changes in practice longitudinally, there was a weak negative correlation between the time (year) of CRS–HIPEC and OS (Pearson correlate coefficient −0.0217, R^2^ 0.047, *p* = 0.015), while there was no correlation between the time of CRS–HIPEC and DFS or LTS. Table 2 demonstrates the COX univariate regression analysis for OS and DFS.

Within our institution, there were 31 patients (24.8%) who survived 5 years or more past their CRS–HIPEC (LTS group) with median OS of 107 months (67–196), and 94 patients (75.2%) who did not (non-LTS group). There were 6 more patients who survived ≥5 years from their carcinomatosis diagnosis, but <5 years from CRS–HIPEC and they were categorized in the non-LTS group. The demographic and tumor characteristics were very similar between the LTS and non-LTS groups, which are summarized in Table 3. The mean age (54.3 vs. 55.2 years, *p* = 0.73) and gender distribution (51.6%, n = 16/31 vs. 50.0%, n = 47/94 male sex, *p* = 0.88) were similar between them as well. Patients had an index colon cancer in 87.1% (n = 27/31) of cases in the LTS group and 86.2% (n = 81/94) of those of the non-LTS patients. There was no statistically significant difference in the disease-free interval between index cancer and peritoneal metastasis (12.9 vs. 10.8 months, *p* = 0.507) or in preoperative systemic therapy use (74.2%, n = 23/31 vs. 83%, n = 78/94, *p* = 0.07) between both groups.

The median PCI was lower in the LTS group (9 vs. 13, *p* = 0.044), and the OR duration was shorter (355 vs. 407 min, *p* = 0.01). Postoperative complication rates were lower in the LTS group (45.2%, n = 14/31 vs. 64.9%, n = 61/94, *p* = 0.04), with Clavien–Dindo grade III and IV complications occurring in 6 patients in the LTS and 16 patients in the non-LTS group. In total, 13/31 patients (41.9%) in the LTS group had disease recurrence post CRS–HIPEC compared to 87/94 patients (92.6%) (*p* < 0.001) in the non-LTS group, with significant difference in the disease-free interval (74.9 vs. 14.2 months, *p* < 0.001). Four patients underwent repeat CRS–HIPEC in the LTS group during the study duration. Treatment and operative details are summarized in Table 4.

Within the LTS group, there were four patients with high PCI (PCI ≥ 20). They had an average OS of 112 months (91.4–141.2), and by the end date of this study, all four had passed away. Their average DFS was 26.8 months (9.1–57.8). All had either well or moderately differentiated colon cancer, with two having synchronous peritoneal disease. Three received preoperative systemic therapy and two had good response on subsequent imaging. The median PCI was 24 (21–27), and CC-0 was achieved in all four cases.

## 4. Discussion

This study is a single high-volume institute study of 125 patients over 15 years that reports long term outcomes and actual survival outcomes of patients with CPC who underwent CRS–HIPEC. Factors associated with worse survival included pre-HIPEC systemic therapy, progression on preoperative systemic therapy, synchronous extraperitoneal metastasis, and high PCI. Factors associated with LTS included lower median PCI and surgery duration, lower incidence of recurrence post CRS–HIPEC, lower rate of postoperative complications and longer recurrence disease-free interval. Multiple other factors that influence survival are described in the literature. Qin et al. (2023) found in their large retrospective review of 371 patients with synchronous CPC that: age >65 years, PCI > 16, use of systemic therapy, CC score, and the use of HIPEC were independent prognostic factors for OS [8]. The use of HIPEC in their population was associated with median OS of 24 months compared to 12 months in CRS alone. Other factors that are described to carry prognostication value for OS in CPC include metachronous onset of peritoneal disease, mucinous adenocarcinoma, differentiation on histopathology, and the presence of extraperitoneal metastasis [9,10].

The role of HIPEC in OS has been questioned more recently following the publication of PRODIGE-7 [7,10], a large, randomized phase III trial which showed no recurrence or survival benefit with the use of HIPEC over CRS alone (Oxaliplatin 460 mg/m^2^ over 30 min after complete cytoreduction) in patients with CPC. In a subgroup analysis, however, it was found that patients with PCI between 10 and 15 seemed to benefit from HIPEC. Additionally, the majority of current HIPEC centers use a different regimen than in PRODIGE-7 (MMC over an hour with lowest temperature of 42 C) [11,12]. Despite these differences, PRODIGE-7 demonstrated the value of complete cytoreduction and systemic therapy, improving the OS to 42 months compared to a dismal prognosis of 10.4–12.5 months with palliative chemotherapy, with only 1.1% of these patients alive in 5 years [10,13]. In this study, 96.8% of the patients had CC-0 and all patients received HIPEC (either with oxaliplatin or MMC). The practice was changed in our center following the publication of PRODIGE-7 to use MMC for 60 min instead of oxaliplatin for HIPEC. The 5-year OS in our population was 24.8% with median survival of 41.6 months. Given the long period of study and changes in practices (newer guidelines, change in HIPEC and systemic therapy regimens, advancement in surgical techniques, and introduction of enhanced recovery after surgery (ERAS) techniques), there was a weak negative correlation between the time of surgery (year) and OS, but no significant correlation was observed with DFS or LTS. The negative correlation could be explained by widened inclusion criteria in later years but also with shorter follow-up duration in more recent surgeries.

The literature around the role of neoadjuvant systemic therapy (NAT) for patients undergoing CRS–HIPEC lacks randomized trials, with large heterogeneity in the retrospective and prospective studies. In their systematic review evaluating the role of NAT in patients with CPC undergoing CRS–HIPEC, Rovers et al. [14] concluded that there is absence of high-level evidence, but despite that, NAT might result in improved survival. Another systematic review concluded the same [15]. The CAIR06 trial is currently in the recruitment phase comparing upfront CRS–HIPEC versus CRS–HIPEC with perioperative systemic therapy [16]. The use of NAT is hypothesized to eradicate micro-metastatic disease, lower the intraperitoneal burden of disease, and test tumor biology [15,17]. In this study, 80.8% underwent NAT, which is part of our standard protocol for patients with CPC prior to CRS–HIPEC. The remaining patients underwent upfront CRS–HIPEC due to systemic therapy toxicity, limited peritoneal disease (median PCI 8) or symptomatology. This could explain why pre-HIPEC systemic therapy was associated with worse OS in our population.

PCI has been shown to be a good prognostic tool and an objective method for patients’ stratification for CRS–HIPEC as it is independently associated with OS and LTS [7,9,10]. The exact categorization of PCI into high or low can be variable according to different centers; however, a PCI ≥ 20 is often considered a mark of extensive peritoneal disease and a relative contra-indication for CRS–HIPEC in some centers [18]. In our population, a lower median PCI was found to be associated with LTS (9 versus 13, *p* = 0.044). PCI, however, remains a subjective estimated evaluation of the burden of peritoneal carcinomatosis. The accuracy of the diagnostic method used to quantify it can differ across centres, with many centres documenting the preoperative PCI with a diagnostic laparoscopy, and others estimating it based on radiological imaging. This can lead to some amount of discrepancy between the intraoperative PCI documented at the time of CRS–HIPEC and the one calculated at the time of diagnosis. Moreover, the response to systemic therapy is often under-evaluated when the PCI is used as a constant variable, and the change in PCI or response to chemotherapy is often not quantified. Other challenges with using PCI as a solo stratification factor for CRS–HIPEC is that most studies often do not include patients who received NAT with planned CRS–HIPEC and were then not offered surgical management due to progression of disease as indicated by worsening PCI, and are often considered as part of the exclusion criteria [19]. The COMBATAC trial [20], which was a multicenter, open-label, single-arm study evaluating patients with CPC and appendiceal carcinomatosis, was the only trial evaluating NAT in the setting of CRS–HIPEC; however, it was terminated due to insufficient accrual. The dropout rate in their population was 36% as these patients progressed on systemic therapy and were considered inoperable. In our population, progression on pre-HIPEC systemic therapy was associated with worse DFS (HR 1.57, 95% CI 1.38–2.81, *p* = 0.043). At our center, we utilize preoperative imaging to analyze the response to systemic therapy to decide about proceeding to CRS–HIPEC. A diagnostic laparoscopy is used selectively as a final decision-point to include or exclude patients from CRS–HIPEC based on the PCI post-systemic therapy (PCI ≥ 20). Further studies evaluating the effect of change in PCI following systemic therapy compared to static intraoperative PCI on OS and DFS are needed.

LTS (defined as patients who survive ≥5 years from the date of CRS–HIPEC) is an area of current literature discussion and evaluation, aiming to identify factors associated with true survival rather than projected OS. A recent study demonstrated 36% actual 5-year OS among 103 patients in a single center in New York [21]. Another study demonstrated similar OS benefit, with 10-year OS of 15%, and 5-year DFS of 16% [18]. Factors associated with improved LTS have included low PCI (<10) and complete cytoreduction [9,19,21], while factors associated with worse LTS have included metachronous onset of CPC and moderate- to high-grade tumor differentiation [9]. There were 31 patients (24.8%) in our population that met the criteria of LTS. Factors identified to be associated with LTS include lower median PCI (9 versus 13), lower surgery duration, lower postoperative complication rates, lower incidence of disease recurrence post CRS–HIPEC and longer recurrence disease-free interval (74.9 months versus 14.2 months). Patients with disease recurrence following CRS–HIPEC represent a challenging subset; however, repeat CRS–HIPEC in some studies is associated with DFS of 9.6 months and median OS of 20–57 months [22,23]. Four patients in the LTS group underwent repeat CRS–HIPEC during the study duration. Another unique subset of patients, identified in our study withing the LTS group, are patients with high PCI. There was a total of four patients with intraoperative PCI of 20 or more, who were found to be within the LTS group with average OS of 112 months. Their favorable OS could be attributed to favorable tumor biology including well differentiation, response to NAT systemic therapy, and complete cytoreduction. The sample is too small however to draw definitive conclusions.

Our study had several limitations. It is a retrospective review with an inherit selection bias for patients to undergo CRS–HIPEC. Moreover, our center is a CRS–HIPEC referral center, and some patients were treated in the center but followed up elsewhere; hence, data availability in some cases was limited. Moreover, given the long duration of the study, practice patterns have changed significantly over time. The use of EPIC, the type of systemic therapy and HIPEC, length of hospital stays and ERAS protocols occurred at different points of time during these years, and it was difficult to account for all practice changes during this period. Not all disease recurrence was biopsy-proven and instead was based on clinical assessment, radiological findings and ancillary pathology. Lastly, CRS–HIPEC outcomes depend on expertise and experience and might not be generalizable.

## 5. Conclusions

CRS–HIPEC remains a valuable option associated with improved OS and DFS in patients with CPC. The 2-year and 5-year OS in our study was 68% and 24.8%, respectively, while the DFS at 2-year was 28.8% and at 5-year, 14.4%. Factors associated with worse survival include high PCI, presence of extraperitoneal metastasis, and disease progression while on systemic therapy. Factors influencing long-term survival (≥5 years) include peritoneal disease burden, perioperative events, and the duration of disease-free interval. Future studies are needed to further assess factors associated with LTS.

## Figures and Tables

**Table 1:** Patients, tumor and peritoneal metastasis characteristics, surgical details, and postoperative course for the entire population (n = 125).

Characteristics	Number of Patients, n (%)
Age, mean (SD), y	54.5 (12.4)
Gender Male Female	63 (50.4) 62 (49.6)
Index surgery	113 (90.4)
Origin of cancer Colon Sigmoid Ascending colon Cecum Transverse colon Descending colon Rectum	108 (86.4) 42 (33.6) 33 (26.4) 20 (16.0) 10 (8.0) 3 (2.4) 17 (13.6)
Index nodal status Positive	82 (65.6)
Pathology Mucinous adenocarcinoma BRAF mutated	35 (28.0) 10 (8.0)
Differentiation Well differentiated Moderately differentiated Poorly differentiated Signet Ring	26 (21.9) 63 (52.9) 22 (18.5) 8 (6.7)
**Peritoneal Metastasis**	
Synchronous	65 (52.0)
Extraperitoneal disease ^1^	22 (17.6)
Preoperative systemic therapy	101 (80.8)
Response to systemic therapy Disease response Stable disease Disease progression	47 (46.5) 40 (39.6) 14 (13.9)
**Surgical Details**	
ASA ^2^, median (range)	2 (1–3)
Complete cytoreduction (CC) CC-0 CC-1 CC-2	121 (96.8) 2 (1.6) 2 (1.6)
PCI ^3^, mean (range)	11 (0–39)
EBL ^4^, mean (range), mL	756 (50–3000)
Surgery duration, mean (SD), min	394.5 (97.2)
Surgical resection Bowel resection Diaphragm stripping Index cancer resection	113 (90.4) 50 (40.0) 24 (19.2)
HIPEC ^5^ regimen Mitomycin C Oxaliplatin	20 (16.0) 105 (84.0)
EPIC ^6^	8 (6.4)
**Postoperative Details**	
Length of stay, mean (range), d	20.9 (7–84)
All complications Clavien–-Dindo III Clavien–Dindo IV Reoperation ICU ^7^ stay	75 (60.0) 19 (25.3) 3 (4.0) 11 (8.8) 21 (16.8)

^1^ Parenchymal liver or ovarian metastasis. ^2^ American Society of Anesthesia classification. ^3^ Peritoneal carcinomatosis index. ^4^ Estimated blood loss. ^5^ Hyperthermic intraperitoneal chemotherapy. ^6^ Early perioperative intraperitoneal chemotherapy. ^7^ Intensive Care Unit.

**Table 2:** COX regression univariate analysis for overall survival (OS) and disease-free survival (DFS) after cytoreductive surgery and hyperthermic intraperitoneal chemotherapy (CRS–HIPEC) for colorectal peritoneal carcinomatosis. Bolds indicate statistically significant value.

Survival	Variable	Hazard Ratio	95% Confidence Interval	*p*-Value
OS DFS	Age	0.99 0.99	0.98–1.01 0.98–1.02	0.492 0.814
OS DFS	Index cancer (ref. colon)	1.18 1.25	0.67–2.08 0.71–2.20	0.566 0.446
OS DFS	Index cancer node-positive	1.06 1.17	0.69–1.62 0.77–1.78	0.801 0.459
OS DFS	Synchronous peritoneal metastasis	0.88 0.95	0.60–1.32 0.64–1.40	0.544 0.787
OS DFS	Synchronous extraperitoneal metastasis	**1.13** 1.01	**1.08–1.90** 0.593–1.69	**0.039** 0.998
OS DFS	Pre-HIPEC systemic therapy	**1.49** 1.53	**1.19–2.49** 0.91–2.58	**0.032** 0.110
OS DFS	Progression on pre-HIPEC systemic therapy	1.07 **1.57**	0.59–1.94 **1.38–2.81**	0.824 **0.043**
OS DFS	PCI ≥ 20	**1.85** 1.34	**1.12–3.06** 0.74–2.43	**0.017** 0.332
OS DFS	Post-HIPEC systemic therapy	1.21 1.38	0.80–1.81 0.89–2.15	0.365 0.154

**Table 3:** The demographic and tumor characteristics of the long-term survival (LTS) group and non-long-term survival (non-LTS) group.

Characteristics	Alive at 5 Years (LTS) 24.8% (n = 31)	Mortality < 5 Years (Non-LTS) 75.2% (n = 94)	*p*-Value
Age (years)	54.28 (±12.82)	55.18 (±11.04)	0.726
Sex n (%) Female Male	15 (48.4) 16 (51.6)	47 (50.0) 47 (50.0)	0.876
Primary tumor location n (%) Colon Rectum	27 (87.1) 4 (12.9)	81 (86.2) 13 (13.8)	0.896
Synchronous peritoneal disease n (%)	14 (45.2)	51 (54.3)	0.379
Synchronous extra-peritoneal disease n (%)	26 (83.9)	77 (81.9)	0.804
Histology n (%) Adenocarcinoma Well differentiated Moderately differentiated Poorly differentiated Signet ring	5 (16.1) 18 (58.1) 4 (13.0) 1 (3.2)	21 (22.3) 45 (47.9) 18 (19.1) 7 (7.4)	0.563
Preoperative systemic therapy n (%)	23 (74.2)	78 (83.0)	0.070
Response to systemic therapy n (%) Improvement of stable disease Disease progression	19 (61.3) 4 (12.9)	68 (72.3) 10 (10.6)	0.577
Index cancer disease-free interval (months) (SD)	12.9 (16.1)	10.8 (15.1)	0.507

**Table 4:** Treatment and operative characteristics of long-term survival (LTS) group and non-long-term survival (non-LTS) group. Bolds indicate statistically significant value.

Characteristics	Alive at 5 Years (LTS) 24.8% (n = 31)	Mortality < 5 Years (Non-LTS) 75.2% (n = 94)	*p*-Value
Type of chemotherapy n (%) Oxaliplatin Mitomycin C	28 (90.3) 3 (9.7)	77 (81.9) 17 (18.1)	0.268
Intraperitoneal chemotherapy n (%) HIPEC HIPEC + EPIC	28 (90.3) 3 (9.7)	89 (94.7) 5 (5.3)	0.390
Median PCI	9	13	**0.044**
PCI group n (%) Low (<20) High (≥20)	27 (87.1) 4 (12.9)	78 (83.0) 16 (17.0)	0.588
CC-0 n (%)	31 (100.0)	90 (95.7)	0.243
OR duration (mins) (SD)	355 (81.2)	407 (98.9)	**0.010**
Complications n (%) Grade 1 and 2 Grade 3 and 4	14 (45.2) 8 (25.8) 6 (19.4)	61 (64.9) 45 (47.9) 16 (17.0)	**0.041**
Postoperative systemic therapy n (%)	9 (30.0)	42 (44.7)	0.155
Recurrence post CRS–HIPEC n (%)	13 (41.9)	87 (92.6)	**<0.001**
Recurrence disease-free interval (months) (SD)	74.9 (52.2)	14.2 (10.4)	**<0.001**

## Data Availability

The datasets presented in this article are not readily available due to institutional and organizational data-sharing restrictions within the province related to patient privacy. Requests to access the datasets should be directed to the corresponding author.
